# Co-design workshops with families experiencing multiple and interacting adversities including parental mental health, substance use, domestic violence, and poverty: intervention principles and insights from mothers, fathers, and young people

**DOI:** 10.1186/s40900-024-00584-0

**Published:** 2024-06-26

**Authors:** Cassey Muir, Sophie G. E. Kedzior, Simon Barrett, Ruth McGovern, Eileen Kaner, Ingrid Wolfe, Julia R. Forman, On behalf of the ORACLE consortium

**Affiliations:** 1 Population Health Sciences Institute, Newcastle University, Newcastle upon Tyne, UK; 2 Department of Women and Children’s Health, King’s College London, London, UK

**Keywords:** Family, Adverse childhood experiences, Parents, Mental health, Children, Young people, Co-design, Co-production, Intervention development, Public involvement and Engagement

## Abstract

**Background:**

Clustering and co-occurring of family adversities, including mental health problems, substance use, domestic violence and abuse, as well as poverty can increase health and behavioural risks for children, which persist throughout the life course. Yet, interventions that acknowledge and account for the complex interactive nature of such risks are limited. This study aimed to develop intervention principles based on reflections from mothers, fathers, and young people who experience multiple and interacting adversities. These principles will show how family members perceive an intervention may bring about positive change and highlight key insights into design and delivery.

**Methods:**

A series of six co-design workshops with mothers, fathers, and young people who experienced multiple and interacting adversities (*n* = 41) were iteratively conducted across two regions in England (London and North-East) by four researchers. Workshop content and co-design activities were informed by advisory groups. Data from facilitator notes and activities were analysed thematically, resulting in a set of intervention principles.

**Results:**

The intervention principles highlighted that: (1) to reduce isolation and loneliness parents and young people wanted to be connected to services, resources, and peer support networks within their local community, particularly by a knowledgeable and friendly community worker; (2) to address feelings of being misunderstood, parents and young people wanted the development of specialised trauma informed training for practitioners and to have the space to build trusting, gradual, and non-stigmatising relationships with practitioners; and (3) to address the needs and strengths of individual family members, mothers, fathers, and young people wanted separate, tailored, and confidential support.

**Conclusions:**

The current study has important implications for practice in supporting families that experience multiple and interacting adversities. The intervention principles from this study share common characteristics with other intervention models currently on offer in the United Kingdom, including social prescribing, but go beyond these to holistically consider the whole families’ needs, environments, and circumstances. There should be particular focus on the child’s as well as the mothers’ and fathers’ needs, independently of the family unit. Further refinement and piloting of the developing intervention are needed.

**Supplementary Information:**

The online version contains supplementary material available at 10.1186/s40900-024-00584-0.

## Abstract (plain language summary)

Families can experience multiple difficulties. These difficulties include parental mental health problems, alcohol and drug use, domestic violence, and poverty. These difficulties can impact the wellbeing of both parents and children. Currently, support that is provided to families rarely accounts for these complex and multiple difficulties. This study aimed to gather insights from mothers, fathers, and young people about how to best support families who experience multiple difficulties at the same time. We ran six workshops with community groups of mothers, fathers, and young people from London and North East England. We learned that: (1) Parents and young people wanted to be connected to services, resources and peer support networks within their local community. (2) Parents and young people wanted to build trusting, gradual, and non-stigmatising relationships with practitioners. (3) Parents and young people wanted support that was personalised to their own needs and that focused on their strengths. This research contributes key ideas for supporting families, which will be used alongside other studies to develop new ways of supporting families. The next steps will be to complete and test the developing support model, by delivering it to families and measuring how well it works.

**Supplementary Information:**

The online version contains supplementary material available at 10.1186/s40900-024-00584-0.

## Background

### Parental risk factors and impact upon children

Adverse childhood experiences (ACEs) are events or circumstances that occur during childhood and are associated with harm, including child abuse and neglect, household dysfunction, parental risk behaviours, and ill health [1, 2]. Parental risk factors may include mental health problems, substance use, and domestic violence and abuse, which increase health and behavioural risks for children throughout the life course [3]. These common risk factors tend to cluster and interact with each other, creating an even more complex and syndemic risk situation for families [4–6]. Across England, it is estimated that 32% of children live in a household where a parent or carer has a mental health problem, 25–27% of children live with an adult who has experienced domestic violence and abuse, and > 10% live with a parent who uses substances [4]. Between 1.3% and 4.3% among children 0–5 years, and 0.7% and 3.2% among children 6–15 years live with someone experiencing all three risk factors [4].

Experience of these risk factors in childhood is associated with negative physical, psychological, social, and economic consequences for children and families [7]. ACEs are understood to have a dose-response relationship, with greater exposure increasing the likelihood of negative outcomes [2]. Individuals who have experienced more than 4 ACEs during childhood or adolescence are at increased odds of health-harming behaviours such as sexual risk taking, substance use, and poor mental and physical health [3, 8, 9]. Beyond behavioural and health outcomes, there is an association between ACEs and lower educational attainment and increased risk of poverty in adulthood [10, 11]. Families living in low-income households are at greater risk of having multiple ACEs compared to high-income households [12]. There is also evidence that poverty is strongly associated with child maltreatment, for example due to family stress and deprivation impacting on the parent’s ability to meet the child’s basic needs [13]. Further, poverty often co-occurs and interacts with other factors, including parental mental health, substance use, and domestic violence and abuse [14], with negative impacts on children’s health outcomes and behaviour in later adolescence [15]. These behaviours and health outcomes can also endure into adulthood, potentially leading to replication of risk factors within their own parenting practices, resulting in intergenerational cycles of adversity [3, 16].

Although there has been increasing evidence of the prevalence and impact on children and families of ACEs [17], little detail is known about the experiences of families dealing with multiple and interacting problems, and how this clustering affects families experiences of support. Preventing problems, identifying children at risk, and providing support at the earliest opportunity could provide long-lasting benefits, and interventions that acknowledge and account for the complex interactive nature of risks are needed [18, 19].

### Interventions addressing multiple adversities

There is currently a lack of interventions that address the complexities and interactions between multiple risks [20, 21], yet the integration and coordination of services is recommended within practice, policy, and research [20, 22, 23]. Most intervention approaches within this field focus on addressing risk factors in isolation. The evidence base is largest for, maternal mental health, but reports limited effectiveness [21]. Interventions rarely address the cumulative impacts of co-occurring risks, or the social factors that may compound adversities for families. Where interventions have addressed multiple needs and had some effect, these tend to offer families sustained support, include connection to community-based services, as well as support parenting capacity and skills [24, 25].

Families experiencing multiple adversities may also require integrated care as adversities cannot be managed by single sectors alone. The integrated care approach stipulates that there is a need for effectively designed services according to the multifaceted needs of the population and the individual/family across their life course [26]. These services are to be delivered by a multidisciplinary team of providers working across settings and levels of care. Alongside these, there is also a need to tackle the upstream causes of poor health, which will lead to the promotion of mental and physical wellbeing [26]. Involving families who experience multiple adversities in the development of interventions and integrated care systems could lead to more effective solutions, as they will be designed based on context-specific, practical, and relevant knowledge of those with lived and living experience.

### Intervention development and co-design

Complex interventions are frequently employed in public health and other health and social care services, which can be implemented and assessed at different levels [27]. Such interventions can range from the individual to the societal level, including a new brief alcohol reduction procedure, the redesign of an integrated health and social care programme, or a change in welfare policy. The Medical Research Council framework for complex interventions recognises intervention development as the first of a series of interconnected steps of the development-evaluation-implementation process [27, 28], but this framework lacks sufficient detail and specificity to inform intervention development. A recently published taxonomy of approaches to developing interventions outlines eight categories to development, one of which is ‘partnership’ [29]. This approach involves active engagement of stakeholders, including the public, in developing interventions, which can facilitate the development of feasible, efficacious, and context-sensitive interventions [30]. Partnership methods can range from consultation to co-design and co-production [31]. We use the definition of co-design for this study, which is the active collaboration among stakeholders relating to solution design, given a pre-determined problem [32]. Through active involvement, we recognise and use the skills, knowledge, and expertise of those with lived and living experience going beyond developing interventions ‘for’ to developing interventions ‘with’ relevant stakeholders [33]. Co-design is an approach that ensures interventions and services reflect the needs and realities of populations they concern [34]. This collaborative approach has shown to improve adaptation and tailoring of interventions and services to be appropriate for a specific context, whilst also identifying the barriers and facilitators critical for intervention success [35, 36]. When co-design is used in the development of interventions, the interventions are more likely to be acceptable, relevant, and focused on changes that are most important to the population they seek to benefit [31]. However, there is a lack of research and clarity about co-designing with families experiencing adversity [37, 38].

This study aimed to develop intervention principles (e.g., broad goals for an intervention) from mothers, fathers, and young people who experience multiple adversities (e.g., parental substance use, mental health, domestic violence and/or poverty). These principles will show how family members perceived an intervention may bring about positive change and highlight key intervention insights (e.g., useful components, design, or delivery of an intervention). We present the processes and findings from a series of co-design workshops undertaken with community groups comprising of mothers, fathers, and young people who have experienced multiple and interacting adversities. The results presented here complement a wider co-produced research project with multiple streams. Together, these streams support evidence-based intervention development for families experiencing multiple and interacting adversities. This approach is in line with previous research that aims to develop an intervention through integration of systematic reviews, qualitative data, and co-design techniques [39].

## Methods

### Study design

The study reported herein forms part of a programme of research (National Institute for Health and Care Research; NIHR200717) to improve outcomes, through preventing or reducing the impact, for children, young people, and families experiencing multiple and interacting adversities. Researchers from King’s College London, Newcastle University, and Liverpool University conducted the research project, ORACLE: OveRcoming Adverse ChiLdhood Experiences. This programme of research aimed to gather data to understand how to support families experiencing multiple and interacting adversities. Findings from four research streams have fed into intervention development that will be integrated to develop an evidence-based intervention for piloting and evaluation. The four research streams are:

- Research stream 1 comprised a qualitative study of in-depth interviews to explore parents and young peoples’ lived and living experiences of multiple and interacting adversities [40].
- Research stream 2 involved secondary data analyses of longitudinal data from the United Kingdom Millennium Cohort study to assess the clustering of trajectories of household poverty and other family adversities and their impacts on adolescent health outcomes [15, 41, 42].
- Research stream 3 involved undertaking a systematic review of reviews and scoping of grey literature to provide an evidence overview of the range and effectiveness of interventions to support children and families where there was multiple and interacting adversities [21].
- Research stream 4, reported here, was informed by the preceding three streams and utilised co-design workshops to explore intervention principles and insights for supporting families experiencing multiple and interacting adversities.

### Ethical approval

Ethical approval for the ORACLE project was acquired from King’s College London Health Faculties Research Ethics Subcommittee (HR/DP-21/22-21189). The workshops were based on a co-design methodology where parents, young people, and researchers held shared ‘power’ in the development of a new intervention, through shared knowledge generation [39, 43]. They were acting as advisors and not participants in the research, and no personal data were collected [43]. Thus informed formal consent to participate in the workshops was not obtained [39]. However, verbal consent was reiterated prior to the workshops commencing and full information on the research process was provided in advance of the workshop by the community organisations or researchers [39]. Ground rules were agreed at the start of each workshop, including respecting each other’s perspectives, acknowledging each person brings value to the discussion, and listening to other’s contributions. Stakeholders were also advised they were able to leave the workshop at any time and did not have to contribute to discussions or activities if they did not want to.

### Public involvement and engagement in study design

Throughout the ORACLE project we have worked closely with the National Children’s Bureau, who have facilitated our consultation with children and parent advisory groups. This public involvement is separate to the co-design workshops (research stream 4). These groups have been involved in key decisions including the initial design of the co-design study, what the key findings of interest to different stakeholder groups could be, and how to present our findings within workshops to different stakeholder groups. The advisory groups therefore supported the development of the focus of the workshops but were not involved in the generation of knowledge about the intervention principles. Members of the advisory groups have also provided feedback on information sheets and ethical procedures. Colleagues at the National Children’s Bureau have also been involved in the monthly ORACLE steering group meetings, ensuring the research remains applicable within practice. This paper follows the GRIPP2 guidelines for reporting public involvement in research [44], see Additional File 1 for the GRIPP2 long form checklist.

### Stakeholders

The researchers identified pre-established peer groups from relevant community-based services. The services were selected to ensure involvement from traditionally under-represented groups, including ethnic minorities, those with care experience, and LGBTQ+. Researchers then organised workshops in collaboration with these gatekeeper service organisations. The researchers had developed strong relationships with these organisations over the course of the ORACLE project. The intention was to involve parents and young people who had self-identified experiences of multiple and interacting adversities and/or who lived in an area of high deprivation. Family members’ expertise and knowledge was related to the experience and impact of living with multiple and interacting adversities and their knowledge on seeking and receiving support and intervention. Parents and young people could also provide new ideas and valuable insights into the potential facilitators and barriers to implementing an intervention to improve outcomes related to parental mental health problems, substance use, domestic violence and abuse, and/or poverty.

There were six workshops in total. For workshops 1 and 2, stakeholders were approached via a London-based charity that supports mothers of young children (0–5 years) living in an area of high deprivation (i.e., an area where 68% of children live in very deprived households). For workshops 3 and 4, stakeholders were approached via a London-based Family Council, consisting of those who had been involved with Children’s Social Care as parents, as well as inviting parents who had taken part in the qualitative interviews from research stream 1. These stakeholders had self-identified lived and living experiences of multiple and interacting adversities. Finally, for workshops 5 and 6, stakeholders were approached via two North-East based charities, one specifically for fathers and the other for children and young people. Both charities support those who live in an area of high deprivation and/or have experience of multiple and interacting adversities.

### Co-design workshop process

Most parents and young people were initially approached by a member of staff at the participating organisations, whilst those that had been involved in the previous ORACLE research stream 1 were approached by the researchers. All were provided with verbal, written, or electronic information about the study and workshops. Those who wanted to be involved came along to one of the workshops, which were scheduled with the support of the participating organisations.

Six co-design workshops took place across two regions in England (London and North-East) from September 2022 to November 2022. Workshops were facilitated by one researcher with support by another, lasting between 90 and 120 min each. This process was iterative with cycles of presenting information and gathering feedback from different stakeholder groups. When workshops occurred on the same day, these were several hours apart, with time for the facilitators to reflect upon and discuss emerging ideas and issues, as well as identify any gaps in knowledge. Most workshops were conducted in person at local community centres, with two workshops (3 and 4) being conducted over video conferencing software to include stakeholders from both regions. To ensure stakeholders felt comfortable and safe to be involved we aimed to hold most of the workshops as separate groups (i.e., mothers, fathers, and young people). For two workshops (3 and 4), we intended to include both mothers and fathers, however only mothers attended these. Such decisions were informed by our project advisory groups consisting of parents and young people.

Within each workshop, stakeholders were introduced to the topic and each other, then three main areas were covered: (1) evidence and information provision; (2) utilising knowledge and experience of stakeholders via workshop activities; and (3) outlining the next steps and gaining feedback.

#### Evidence and information provision

Key findings from research streams 1–3 of the ORACLE project informed the workshops at appropriate points. For example, interventions identified in the review of reviews and scoping exercise (research stream 3) were initially tabulated by the research group, then short summaries with graphics were developed for each and taken to workshops 1–4. These were user friendly and in an engaging format where stakeholders could consider and discuss barriers and facilitators of each. Possible universal interventions were home visiting and regular parental wellbeing checks; routine enquiry and screening for trauma that parents were exposed to as children; coaching or advocacy; conditional and unconditional cash transfers; and youth work. Possible risk factor-specific interventions were therapy or counselling; psychoeducational training; and integrated parental programmes with a risk factor focus. These interventions provided a useful and evidence-based framework for prompting discussion but did not limit what interventions could be discussed, including for instance prevention focused interventions.

Scenarios were also developed by the researchers, with support from the advisory groups. The scenarios were used within workshops that communicated findings from the qualitative data (research stream 1) about lived experiences and impacts of multiple and interacting adversities, e.g., the impact of social isolation. Scenarios use storytelling to explore intervention design ideas by grounding them in context and lived experiences [45]. For example, one of the scenarios focused on a mother with a young child who wanted support for their own mental health, their partner’s substance use, and their child’s wellbeing. The mother had been struggling with feeling isolated and alone in her experiences and was not sure where or how to get support. She visited her general health practitioner to talk through options. Stakeholders were then able to discuss possible solutions and priorities for different family members using the scenarios. Also, by focusing on findings and scenarios from the interviews, stakeholders did not have to share personal experiences. Within the workshops with parents, we also shared simplified findings about the impact of adversity on families for example, 50% of children in the United Kingdom (UK) experience poverty and poor parental mental health (research stream 2). Verbal summaries of discussions and updates on developments since the earlier workshops were also provided.

#### Utilising knowledge and experience of stakeholders via workshop activities

Different approaches to the workshop activities were informed by consultations with our project advisory groups consisting of parents and young people. For instance, parents preferred group-based discussions whereas young people preferred creative activities, including drawing. Table 1 documents the content of each workshop.

**Table 1:** Summary of the workshop content and co-design activities

Workshop No.	Stakeholders	Content and Co-design activities
1 2	12 mothers 5 mothers	Both workshops were held on the same day at a community centre based in London and followed the same format. Stakeholders were guided to share insights and ideas around concerns and improvements or alternatives across possible universal interventions, identified from research stream 3. Stakeholders explored which interventions were acceptable, why, and how they could be improved, through a traffic light system activity. They also shared insights into solutions to any barriers identified.
3 4	5 mothers 2 mothers	Both workshops were held remotely on the same day using video conferencing software and followed the same format. Stakeholders were guided to share insights and ideas on risk factor-specific interventions for mental health, substance use, domestic violence, and poverty, identified from research stream 3. They explored which interventions were acceptable, why, and how they could be improved through group discussion. Stakeholders discussed common principles for interventions and barriers, and issues with resourcing of existing services. They also shared insights into solutions to the barriers identified.
5	6 fathers	This workshop was held at a community centre based in the North-East. Through group discussion, stakeholders identified current support benefits as well as barriers and issues with existing services and interventions as discussed in previous workshops. They shared ideas on how to tailor services/interventions around inclusivity for fathers and how they would experience an intervention.
6	11 young people (aged 13–18 years)	This workshop was held at a community centre based in the North-East. Through group discussion, stakeholders identified current support benefits as well as barriers and issues with existing services and interventions as discussed in previous workshops. They shared ideas on how to tailor services/interventions around inclusivity for children and young people through the development of personas for a service provider and the young people who may use a service. They utilised the personas to map out the experience of a young person going through a service and their interactions with a service provider.

**Fig. 1:**
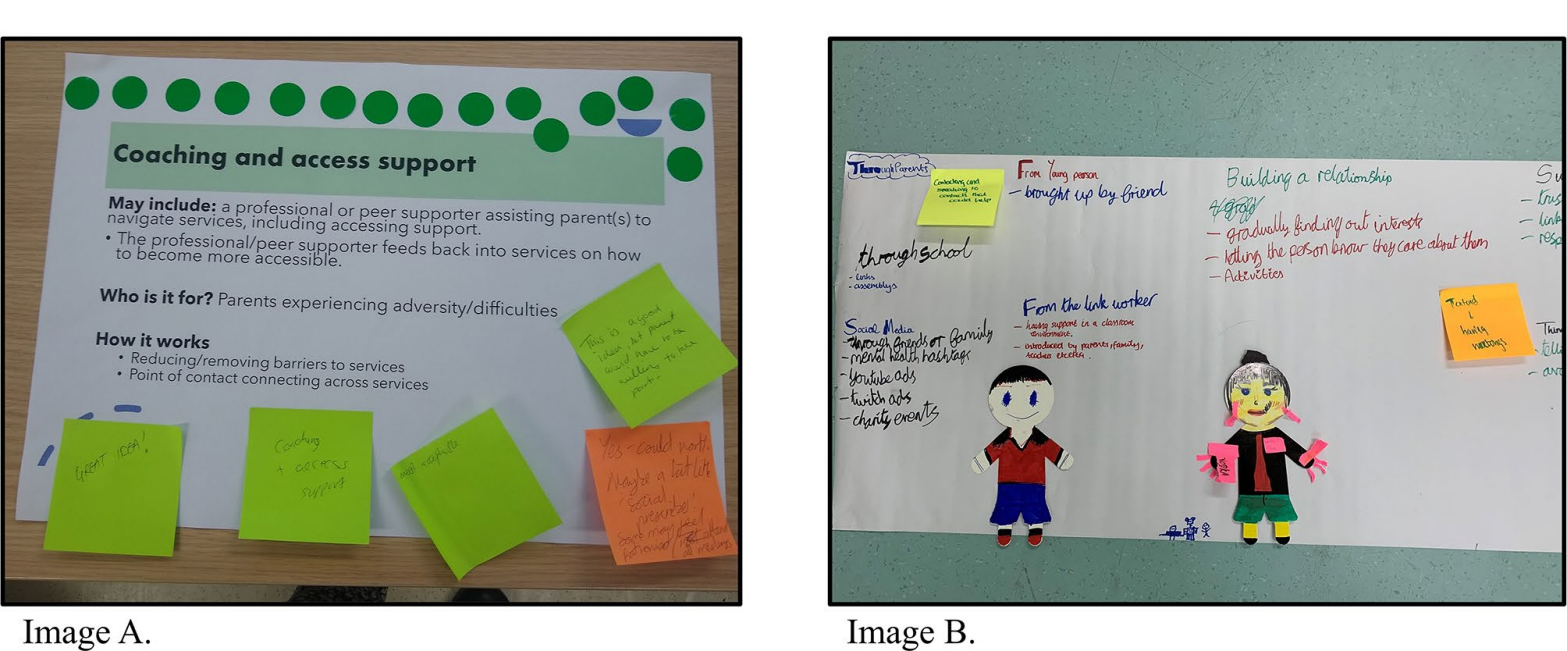
Images from the co-design workshops. Image **A** depicts the traffic light activity in workshop one. Image **B** depicts the journey mapping activity in workshop six

Within workshops 1–4, stakeholders were guided to prioritise the interventions identified from research stream 3 from most acceptable (green), somewhat acceptable (amber), to least acceptable (red) by individually placing coloured ‘sticky notes’ with their reasoning on the different interventions or through group discussion (see Fig. 1, Image A, for an example). Stakeholders were also guided to provide input on acceptability, how to adapt an intervention, as well as barriers and facilitators to implementation. Stakeholders could also discuss and prioritise any other intervention for supporting families. Using the scenarios (e.g., the mother with a young child) helped to focus discussions on what might work, for whom and why. Workshops 5 and 6 further explored what interventions would be acceptable for fathers as well as children and young people and how to tailor interventions to meet their needs.

As workshop 6 involved young people, we utilised creative activities, including developing personas and drawings of a service user and service provider. Personas are fictional characters used to represent typical users of an intervention [46]. For example, young people discussed that a service user may be a teenager who needs support with their own substance use and mental health problems, separate from their family. They drew this young person and wrote notes on the back regarding their persona’s characteristics and experiences, for example, “getting into trouble at school” and “no support at home”. They also wrote, drew, and discussed characteristics of what a good service provider was, for example they drew big ears for a good listener and a big heart for being kind and caring. The service provider could be anyone they thought would support young people. Using the personas and drawings developed by the young people, they were guided to map the journey of the service user (i.e., a young person) through an intervention that they thought would be acceptable and useful for young people who experience multiple and interacting adversities (see Fig. 1, Image B, for an example). The group focused on a link worker type intervention, where young people could access lots of different support options from a single practitioner. They discussed and mapped out how a young person would find out about the intervention, how they would build a relationship with the practitioner, how the young person would be supported, and what the outcome or ending of the intervention would look like.

Across all workshops, discussions included how poverty influences families’ access and engagement with current interventions and support, and how to address multiple and interacting adversities within interventions. We also discussed strategies for including fathers and children directly within interventions, as most current interventions targeted mothers. Whilst we explicitly involved fathers and young people in our workshops to explore their views, we also explored mothers’ views on how to include fathers and children in interventions typically aimed at mothers. Furthermore, we discussed how to address the social exclusion and isolation families experience.

#### Outlining the next steps and gaining feedback

At each workshop verbal summaries of the workshop and next steps were provided and stakeholders were given the opportunity to ask questions and provide feedback either in group discussions or privately. Each stakeholder was provided with a voucher for their involvement, in line with national standards for public involvement and engagement [47]. After each workshop, the researchers had wider team meetings to reflect on the workshops, intervention insights, and the process taken.

### Data collection and analysis

Data were captured in written form, through facilitators’ detailed notes and summaries during and after each workshop to capture discussions and key insights about intervention development. Both verbatim quotes from stakeholders and facilitator interpretations were captured. Staff from the gatekeeping organisation for workshop 1 and 2 also provided notes that captured the discussions. All completed activities were also utilised as data (e.g., written notes from stakeholders). A pragmatic approach informed by reflexive thematic analysis was applied to the workshop findings [48]. Data, including facilitator notes, captured quotes, and stakeholder notes were tabulated from each workshop. Descriptive codes were initially identified, which stayed close to the participants’ meanings and quotes, and then latent codes and themes were identified through team discussions, that reflected our interpretations and inferences of the descriptive codes. Recurring codes and themes were sought and grouped across workshops and refined within research team meetings to produce meaning-based themes [49]. These themes became the ‘intervention principles’ detailing the insights from mothers, fathers, and young people who experience multiple and interacting adversities. To account for biases from the research team, who have varied professional experience but limited ethnic and gender diversity, we presented developing principles at subsequent workshops (e.g., taking a trauma informed approach) for feedback and sense-checking. Whilst stakeholders were not involved in writing up the results, we consulted with the advisory groups on the presentation of the results.

## Results

### Stakeholder characteristics

Forty-one stakeholders were involved across six co-design workshops. There were 24 mothers involved over four workshops: workshop one (*n* = 12), workshop two (*n* = 5), workshop three (*n* = 5), and workshop four (*n* = 2). Workshop five (*n* = 6) comprised of fathers and workshop six (*n* = 11) comprised of young people aged 13–18 years. No other demographic details were collected.

### Intervention principles

The intervention principles identified for families experiencing multiple and interacting adversities are outlined below. These principles provide insights into the ways in which mothers, fathers, and young people perceived an intervention may bring about positive change and are organised under three themes: (1) building a network to reduce isolation; (2) establishing a trauma-informed approach; and (3) tailoring support to the family’s needs and focusing on their strengths.

#### Building a network to reduce isolation

*“Support is out there but we don’t know about it”.* Approaches which aim to reduce the isolation felt by parents and young people who experience multiple and interacting adversities were thought to be important. Building a network was identified as a possible solution to address such feelings and experiences of loneliness. This could be achieved through either increasing awareness of community services through a specified practitioner and/or providing access to peer support. Parents and young people recognised that there were already many different support services and groups in their local area targeting the different risk factors or with an aim to reduce isolation and loneliness, for instance group support for substance use. However, the problem was that they did not know what options were available to them, how to find out about them, or how to access them. Within formal appointments with healthcare practitioners, parents felt that appointment times were not long enough to discuss additional support needs such as meeting people in similar situations to themselves, what services were available to them, or their concerns over issues relating to poverty. Moreover, finding local services without the help of practitioners could be confusing and challenging. Parents and young people across the different workshops highlighted the importance of peer support, community, and informal contacts and networks as a key strategy of knowing which services and opportunities are available to them. Many shared that developing a formal approach to identify the right support would be useful, including an informational booklet, a digital application, or central contact person. Based on parents and young people’s preference across the workshops, conversations mainly focused on a specified contact person.

Mothers with lived experience of multiple and interacting adversities shared the insight that they wanted a person in the role of a *“companion, buddy, or befriending service”* who could support access into different services and provide emotional support throughout that journey. Some mothers had found this type of support from HomeStart or their Children’s Centre but this was not typical for all. The principle was that the supporter could initially accompany them to services, but over time help the parent develop skills, strategies, and confidence, to empower them to attend services or meetings independently in the future. Likewise, fathers spoke of wanting someone to *“walk alongside”* them during the process of being referred onto and waiting to attend different services, as they had experienced anxiety of being moved through different services without support. Young people also stated that they would like to have a *“buffer person”* who could support them to access and engage with specialised support or interventions (e.g., Children and Adolescent Mental Health Services). This person was thought to help young people overcome social anxiety and lack of self-confidence when accessing services on their own, as well as being a friendly person to rely on during waitlist times. This person would be knowledgeable about available support services and be able to link them to support, rather than attempting to find support themselves.

For parents (both mothers and fathers), peers with lived experience of family adversities were discussed as important to help them navigate access to support, which also provided opportunity for shared understanding and empathy. They felt that talking to someone who had similar experiences to themselves would help them overcome the fear of judgement and stigma often felt when in formal consultations with practitioners. The opportunity to meet peers could also encourage hope and aspirations, as stories could be inspirational, wherein parents could learn from their peers *“navigation of the support system”*. Fathers found peer support as an important element of their journey as they were able to talk and create connections with dads in similar circumstances, often feeling left out and isolated from formal support services. Peer support may also be useful for young people, but they felt that this would only work in a controlled environment with strict rules about disclosure and confidentiality. Young people emphasised that peer support would mainly be beneficial if the peer mentor was going along to different activities with them.

#### Establishing a trauma-informed approach

*“We need to feel understood”.* Approaches which aim to address and understand the trauma experienced by parents and young people living with multiple and interacting adversities were perceived to be important. Parents and young people wanted their experiences of mental health, substance use, domestic violence and abuse, and/or poverty to be understood by the practitioners they encounter, whether within health and social care or voluntary and community sector organisations. Parents and young people provided the insight that specialised training for practitioners would be useful within current support and practice, as well as having time to slowly build trusting relationships with practitioners. Both solutions were thought to have the potential to address parents and young people’s feelings of being misunderstood, and issues with trauma and stigma.

Many parents shared concerns around disclosing mental health problems, substance use, or domestic violence during appointments with practitioners. This was often due to the fear and stigma of how the practitioner might respond and of child protection services involvement, or previously experiencing discrimination from practitioners, impacting their self-esteem. For instance, parents discussed that if a parent is told by a healthcare practitioner that they will not be provided with mental health support until they have reduced their substance use this can be a highly stigmatising and traumatic experience for the individual, especially if not handled in an understanding and sensitive way by the practitioner. They felt that parents would need to be supported concurrently with their differing needs, with joined up services for individual risk factors (e.g., mental health and substance use services) or a bridging practitioner between the different services. At the least, parents discussed that they wanted practitioners to understand the lived experiences of parents with multiple and interacting adversities. Furthermore, parents believed that practitioners needed to be aware of the possibility of retraumatising the parent by directly and bluntly asking about their childhoods, experienced violence, mental health, or substance use within appointments. There needed to be sufficient external support in place if this was the case. Fathers indicated that direct questions about their mental health made them lose engagement quickly, but questions such as *“How are things at home and at work?”* facilitated conversations more effectively and felt less threatening. Young people also thought it was important to first discuss things other than mental health or problems at home, like talking about hobbies, TV, or shared interests. Whilst parents thought it could be useful to ask about financial situations in healthcare appointments, they thought it could be stigmatising and that practitioners would need to ask this question in a non-threatening way e.g., *“do you ever struggle to make ends meet?”* The characteristics of the practitioner and their questioning were therefore crucial in how parents and young people engaged with support; they needed to be sensitive and tactful. Parents and young people felt that training for practitioners could build the confidence of the practitioner whilst also improving the practitioner-client relationship and the families experience of support. It was discussed that such training could include, awareness raising that there is an interaction between different family adversities and the lived experience of poverty, examples of how to respond compassionately through changes to language and behaviours and how to ask questions appropriately, as well as how to resist re-traumatisation of parents and young people.

Parents and young people reported preferring relational interactions and a graduated trust building approach to support. However, within short appointment times, it was difficult to develop such a trusting relationship with a health or social-care practitioner. Therefore, there was a need for an honest, transparent, and understanding practitioner, who may be external to their standard health or social care appointments, with whom they could slowly build a relationship. This approach was thought to include talking to a practitioner about things that were not directly related to support or their problems and participating in activities together like having a coffee or going for a walk, which helped to build trusting relationships and *“broke the ice”*. This gradual process allowed families to feel more connected to the practitioner and able to be open about their experiences and what support they needed. Continuity in the person providing support was also important, as parents and young people did not want to have to repeatedly tell their stories to different people, as this could also be traumatising.

#### Tailoring support to the family’s needs and focusing on their strengths

*“We need support for each family member”.* Approaches which aim to address the needs and understand the strengths of different family members were important. Mothers, fathers, and young people all discussed how current services may not be as inclusive to their individual needs as they would have liked. Mothers wanted to be provided with opportunities that focused on their individual needs and strengths, and not on them as a mother, including finding opportunities to pursue passions such as art or outdoor activities. Mothers also felt that services should be separate for different family members, linking up only if or when needed, allowing for privacy. Separate support was also discussed as crucial in the case of disclosed or undisclosed domestic violence and abuse within the family. Fathers acknowledged that services were not typically focused on, inclusive of, or accessible to dads. Whilst fathers recognised that the forms of support that they would need were similar to that of mothers, they often felt excluded from informational leaflets, appointments, and discussions, which were usually targeted towards the mother. As an example, they cited midwives, where they felt additional training could ensure that they consider the needs of the father and recognise that the whole family needs support, not just the mother and child. Fathers wanted to be included in appointments, either together with other family members or separately but with an effort from practitioners to understand their perspective. Where services include multiple family members, particular sensitivity and care will be needed around domestic violence and abuse.

Similarly, young people discussed that practitioners who are supporting parents could also seek to identify whether their children, especially of adolescent age, may also need their own support as well. Young people felt confidentiality or lack thereof was a major issue regarding support that included other family members. They wanted support separate from their parents, where they felt respected and acknowledged as their own person. Having support tailored to their own needs, rather than the needs of the family, would allow young people to feel safe and not fear reprisals or increased problems if issues were disclosed in front of their parents. There was a felt conflict between practitioners being able to safeguard the child whilst also providing them with confidential support. Young people felt that when appropriate, practitioners should involve young people directly in decisions about their care so that they know what is happening and can prepare themselves. This was especially important if that meant a practitioner needed to tell parents about what a young person discussed in their appointment. Young people also felt that support should be age-appropriate and tailored to be inclusive of those from different backgrounds, cultures, or genders. Schools were also identified as a place where young people could potentially access support, as it is separate from the family. However, there is significant variation in schools, and particularly in their resources to support young people in this way. In addition, it was important that support within school did not further stigmatise or single out young people, for example, being taken out in the middle of a class for support was viewed as embarrassing.

All parents and young people expressed the need for flexibility and adaptability of an intervention, where they could choose when they access (e.g., weekday/weekend, day/evening), how they access (e.g., phone call, text, in person) or where a service is situated (e.g., healthcare, community, home, school). It was felt that the intensity and frequency of support being provided needed to be varied and tailored to different family members. Being provided with such choices, as well as being supported to develop their own goals and skills was thought to help empower families.

Poverty was seen as a barrier to accessing support and impacted on the whole family’s wellbeing, but parents felt there was not a *“quick fix approach”* to addressing poverty issues. A tailored approach that suited the needs of the family and their financial situation was thought to be of most use to parents. Being provided with financial advice and information by a practitioner, or having referrals made into services to address welfare or housing issues were discussed as being useful by parents. Parents also wanted to be offered choices so they could decide what would be of most benefit to them financially, including being offered cheaper or discounted extracurricular activities, being provided with vouchers for food, or being provided with vouchers specifically for their children. Where parents are provided with an unconditional cash transfer, this was thought to be unsustainable, as parents would need other forms of support in conjunction. Importantly, the provision of free childcare or creche support when accessing services was also discussed as necessary for parents of young children, which could allow them to access support and gives them time and space to process and reflect on the session before stepping back into childcare responsibilities.

## Discussion

The current research explores insights of mothers, fathers, and young people who experience parental substance use, mental health, domestic violence and/or poverty in order to establish intervention principles that would guide intervention development. Previous co-design studies [50–52] and qualitative studies [53–55] have typically focussed on a single risk factor or only explored insights from one family member group (e.g., mothers or children). Whilst such studies provide an important understanding of experience and impact, they lack the understanding of how these risks often cluster and exacerbate adversity, as well as how to address support within the whole family.

Co-design workshops with parents and young people produced three principles that detail the ways in which these stakeholders perceived an intervention may bring about positive change for families experiencing multiple and interacting adversities. These include building a network to reduce isolation; establishing a trauma-informed approach; and tailoring support to the family’s needs and focusing on their strengths. The findings highlight that families with multiple and interacting adversities need a package of care that integrates all the principles, which may be challenging due to the fragmentation or siloed nature of current service provision. However, these principles point towards interventions where there is already a growing evidence base, and which share similar characteristics. For instance social prescribing [56], home visiting [57], and peer models of support [58] could potentially be adapted and built upon for families experiencing multiple and interacting adversities. Similarly, within Australia, researchers are co-designing an integrated community healthcare hub with and for families who experience adversities to effectively use service resources [59, 60]. Co-design could improve implementation despite issues with fragmented services, competing pressures, and reduced budgets due to the person-centred perspective and real-world discussion during intervention development. It could make the system more efficient, trustworthy, and responsive to community needs, whilst also empowering those who have helped develop it [61]. Understanding how families use and could improve upon or design interventions may help to produce acceptable and effective insights that translate into practice [62]. In the following sections, we mainly focus on comparing the intervention principles reported here with the characteristics of similar models.

The parents and young people in this study identified the importance of alleviating loneliness and isolation mainly through the establishment of a formal community health worker style service that could provide holistic emotional and practical support for the whole family in a way that met their individual and family needs. This support would include linking to services specifically for substance use, domestic violence and/or mental health, as well as social and skill building activities and groups. Such an intervention was thought to help families to become integrated into the community, provide greater access to formal and informal services, and improve parent and child health and wellbeing, with a worker who acted like a family friend.

This approach has similarities to social prescribing, which is a service within primary care involving a link worker attending to the non-medical needs of service users by linking them to community and voluntary services and additional resources [56]. A link worker is typically a non-health or social care professional with an extensive knowledge of local community resources [56]. The community resources people are linked up with can improve health-related behaviours and social interactions of people as well as reduce demand on primary and secondary care services [56, 63, 64]. The principles within our study expand on this approach, to ensure the needs of the whole family, and not just the presenting individual, would be met, by working with the individual to identify other family members who may need their own tailored support (e.g., a father or child). This also contrasts with family systems approaches, which often focus on the family functioning as a whole to improve children’s wellbeing [65] whilst overlooking individual needs. However, there is a need to consider the sensitivity of the context and appropriateness of these approaches, for example in the context of domestic violence and abuse.

Service users of social prescribing services have reported that the strong and supportive relationship with an accessible link worker was an important aspect of their experience [66]. Similarly, within our study, the development of trusting relationships was viewed as a key enabler for parents and young people as it could help reduce feelings of stigma or shame. Overcoming stigma is an important factor in providing support for parents and children who experience parental substance use, mental health, domestic violence and/or poverty [67–69]. Several qualities were considered essential to good quality and non-stigmatising support, which should be taken into consideration for future interventions. These include the need for ongoing support with a limited number of practitioners to establish trust and a sense of safety, for support to feel informal whereby the relationship-building element is more important initially than addressing the risk factors, and the use of peer mentors or groups of those in similar situations. Many of these attributes reflect previous research, especially regarding trauma-informed care [67, 70, 71] and peer mentoring interventions whereby establishing positive social connections can strengthen resilience and provide emotional and practical support [51, 58, 72]. Similarly, within an Australian context for prevention of mental health problems amongst families experiencing adversities, intervention priorities were those that enabled them to build trusting relationships with service providers and increased their knowledge about available support [73, 74].

Poverty has been identified as a key reinforcing factor in the experiences of families living with multiple and interacting adversities and is associated with double the odds of poor childhood outcomes [15]. Therefore, to identify families experiencing multiple and interacting adversities who may benefit from social and practical support, it is important for practitioners to understand and assess family’s experiences of poverty. Parents within this study highlighted that issues surrounding poverty would need to be approached in a non-threatening way, yet poverty issues are not currently routinely asked in health-care appointments within the UK [75]. Due to the focus upon families in this study and from parents’ insights, there may be potential to pilot an initial assessment question around poverty within midwifery services for expectant parents and their families, to identify need for additional support. A single question approach has been developed in Canada for primary-care practitioners, mainly using the question *“Do you ever have difficulty making ends meet at the end of the month?”*, which identifies patients that would benefit from accessing resources and benefits for which they are eligible [76]. However, this screening tool is not without some implementation barriers, including escalating high workload of practitioners, fear of awkwardness in consultations, and a fear of helplessness without an adequate intervention to help patients out of poverty [75, 76]. Further research would need to address, pilot and test whether such an approach would be acceptable and feasible within midwife appointments with parents.

Within the UK there are currently several social prescribing models that are often targeted at people in socioeconomically deprived areas [66]. However, to date few social prescribing services have tried to address the wider economic issues, whereby support workers provide advice and information, or make referrals into services to address debt, welfare, employment, and housing issues [56]. Where some have tried to address this, such as the Deep End programme in Glasgow that incorporated financial advice and support services particularly in areas of high deprivation, issues with implementation have been reported [77]. Interventions including income supplementation and access to welfare benefits have also been found to reduce a variety of adverse childhood experiences and their impacts [25, 78], yet support services and interventions rarely engage with the impact of income, employment, and housing conditions on families [79]. There is a need to expand on these models, by ensuring families have both social and financial support, with genuine financial options or choices provided and that information provision and support is tailored to the family’s needs.

From a policy perspective, preventing and responding to multiple and interacting adversities requires a system-wide approach that integrates and coordinates efforts across sectors, services, and platforms such as maternal and child health services, education, and welfare policy. There is a need for further funding into community hubs that provide an access point for families to reach services they need, including those mentioned above and which are also tailored and co-designed with mothers, fathers, and young people separately and together. Furthermore, policies that focus on providing income supplementation may not be solely appropriate and effective for families experiencing multiple adversities and should be implemented in a tailored approach alongside relational support that meets the needs of the family.

### Public involvement

Involvement of members of the public was key to this work and supported by the UK health research funding bodies policies, which require public and patient involvement and engagement throughout their funded studies. We developed this study based in line with policy requirements of our funders and ensured meaningful involvement and contribution by working closely with gatekeeping organisations and public partners. The public, comprising parents and young people, were involved in this study via two routes. The first route was via advisory groups whereby the public were involved in study design and the second route was via co-design workshops whereby the public were involved in intervention development. The advisory groups were more general, with public members not necessarily having lived experience of the risk factors addressed here, whereas the co-design workshops mostly involved those with lived and living experience. The advisory groups had knowledge and expertise of research processes, which were invaluable for providing feedback, ideas, and advice on the study design and how to engage certain groups. However, there would have also been value in convening an advisory group of those with lived and living experience, who could assist on topic specific research decisions for example, advising on what language to use when discussing poverty and adversities as well as practical issues of involvement for families experiencing multiple adversities. We suggest other researchers aim to have a mix of public advisors, those with experience of being involved in research and those with lived and living experience, as well as providing training on research skills to those new to public advisory groups.

### Strengths & limitations

To our knowledge, this is the first study that utilised an iterative co-design approach to gain intervention development insights for supporting families who experience parental substance use, mental health, domestic violence, and poverty. Our study demonstrates that families who experience multiple and interacting adversities and who are typically referred to as ‘hard-to-engage’ can effectively be involved and engaged in research, providing meaningful contributions to intervention development.

We built upon our formative research in this area to translate those findings into co-design workshops. Workshop content and style were informed by a national charity and their public advisory groups on how best to engage with parents and young people. Recruited stakeholders represented different family members, working with mothers, fathers, and young people who experience or are affected by multiple and interacting adversities from two regions of the UK (London and North-East). A concerted effort was made to engage with fathers and children, as these individuals are often underrepresented in support directed to the family within this area [21], however we did not manage to recruit members from the same family. Furthermore, although we managed to recruit fathers in the workshop specifically for fathers, we did not manage to recruit fathers into the parent workshops. This finding indicates that future co-design research in this area should strive to hold more father-only focused groups. A limitation of the study is that the parents and young people were already engaged in services. Whilst their views are important, these individuals may have different perspectives on the value of support, barriers to access, and what that support should look like compared to parents and young people who are not engaged in services.

We did not evaluate the impact of public involvement in our research, but feedback gathered after the workshops from parents, young people, and gatekeepers was that the research was important, and that they were happy to be positively contributing to developing support for other families. Through working with the gatekeeping organisations and not collecting personal data, it was possible that there were stakeholders involved who may not have had direct lived experience of any of the risk factors. These stakeholders would however have knowledge of accessing universal family support services and could share their knowledge and experience of such. This is particularly relevant for understanding possible routes to identification or referral of those who would benefit from the co-designed intervention.

Within this study we did not engage with any policy and practice practitioners, who could have provided further valuable insight into the acceptability and feasibility of an intervention. However, we will include potential service providers and policy makers in future intervention refinement phases. Nonetheless, through engaging with local charities there were practitioners and service providers in attendance at the workshops, who expressed their interest in the developing ideas of family members. These practitioners however did not contribute to the discussions and knowledge generation during the workshop.

The sample within this study is also limited by those who were prepared to participate within workshops and group activities. We also recruited previous participants from the formative research streams, whilst this enabled us to sense-check findings, we may have over-represented certain views. Group approaches to workshops may be challenging, due to socially desirable responses or not all voices being heard. However, we aimed to ensure all activities could be done individually or as a group, with the facilitators also making time to speak to each public member individually, especially if they had not contributed as much. We also engaged with established community groups, meaning workshop members already knew each other well, helping with the group dynamics. Data on possibly important variables that may have an influence on perceptions of interventions for those who experience multiple and interacting adversities were not collected, including ethnicity. However, we engaged with community groups who had members from minority ethnicities as well as those receiving support relating to their gender or sexuality.

### Next steps

We aim to continue to work together with relevant stakeholders, especially seeking involvement of practice and policy practitioners, to co-produce and refine an intervention for families who experience multiple and interacting adversities. We also seek to include family members from the same family, where possible, to ensure the resulting intervention meets their needs and to understand identification of family members, as well as acceptability and confidentiality issues. Drawing on the intervention principles from this research, further work would be required to finalise an evidence-based intervention as well as pilot, test, and evaluate it. Moreover, existing interventions could incorporate some or all of the principles that have come out of this work. Parents and young people should continue to be involved in future co-design and intervention development work within this area, using accessible and appropriate engagement methods for different stakeholder groups.

## Conclusions

The current study has important implications for practice in supporting families facing multiple and interacting adversities. Our findings demonstrate that families want support that appropriately addresses the relationships between mental health, substance use, domestic violence and/or poverty, whilst also acknowledging the strengths of families. Parents and young people require support that reduces feelings of loneliness and isolation through ensuring families are formally supported to access social and economic advice, services, resources, and peer networks that can be of benefit to them, but that they are not currently aware of. Trust and safety are important factors in developing non-stigmatising relationships with practitioners and services, which may be easier to achieve with practitioners in an informal environment and who have time to gradually build a relationship with them rather than within healthcare appointments. Families want support that can be offered to the whole family, but where mothers, fathers, and children can have support in their own right and which can be tailored to their needs and strengths. The intervention principles from this study share common characteristics with other intervention models currently on offer in the United Kingdom, including social prescribing, but go beyond these to holistically consider the whole families’ needs, environments, and circumstances. There should be particular focus on the child’s as well as the mothers’ and fathers’ needs, independently of the family unit. Further refinement and piloting of the developing intervention are needed.

### Electronic supplementary material

Below is the link to the electronic supplementary material.

Supplementary Material 1

## Data Availability

The datasets used and/or analysed during the current study are available from the corresponding author on reasonable request.

## Abbreviations

UK: United Kingdom
ACEs: Adverse Childhood Experiences
ORACLE: OveRcoming Adverse ChiLdhood Experiences Research Project
