# Supplementary material for: Co-design workshops with families experiencing multiple and interacting adversities including parental mental health, substance use, domestic violence, and poverty: intervention principles and insights from mothers, fathers, and young people
Source: Res Involv Engagem. 2024 Jun 26;10:67. doi: 10.1186/s40900-024-00584-0 (PMC11202333; doi:10.1186/s40900-024-00584-0)
Supplement: Supplementary file 1 — Supplementary Material 1 [file 40900_2024_584_MOESM1_ESM.pdf]

GRIPP2 Long Form Checklist

Co-design workshops with families experiencing multiple and interacting adversities including parental mental health, substance use, domestic violence, and poverty: intervention principles and insights from mothers, fathers, and young people

Section and Topic	Item	Reported on Page Number
Section 1: Abstract of Paper		
1a. Aims	Report the aim of the study relating to PPI.	Page 2
1b. Methods	Describe the methods used by which patients and the public were involved.	Page 2
1c. Results	Report the impacts and outcomes of PPI in the study.	Page 2
1d. Conclusions	Summarise the main conclusions relating to PPI in the study.	Page 3
1e. Keywords	Include PPI, 'patient and public involvement' or alternative terms as keywords.	Page 3
Section 2: Background to Paper		
2a Definition	Report the definition of PPI used in the study, including any rationale, and how it links to comparable studies.	Page 7
2b. Theoretical underpinnings	Report the theoretical rationale and any theoretical influences relating to PPI in the study.	Page 7-8
2c. Concepts and theory development	Report any conceptual or theoretical models, or influences, used in the study.	Page 7-8
Section 3: Aims of Paper		
3. Aims	Report the aim of the study (in relation to PPI).	Pages 8
Section 4: Methods of Paper		
4a. Design	Provide a clear description of methods by which patients and the public were involved.	Pages 10-18
4b. People involved	Provide a description of patients, carers and the public involved with the PPI activity in the study.	Pages 10-11
4c. Stages of involvement	Report on how PPI is used at different stages of the study (e.g., Identifying, and prioritising research ideas, designing and managing research, undertaking the research, and dissemination and implementation of research).	Pages 10-18

**4d. Level or nature of involvement**	**Report the level or nature of PPI used at various stages of the study (e.g., some people aim for a collaborative approach).**	**Pages 10-18**
**Section 5: Capture or measurement of PPI impact**		
**5a. Qualitative evidence of impact**	**If applicable, report the method used to qualitatively explore the impact of PPI in the study.**	**N/A**
**5b. Quantitative evidence of impact**	**If applicable, report the method used to quantitatively measure or assess the impact of PPI.**	**N/A**
**5c. Robustness of measure**	**If applicable, report the rigour of the method used to capture or measure the impact of PPI.**	**N/A**
**Section 6: Economic Assessment**		
**6. Economic Assessment**	**If applicable, report the method used for an economic assessment of PPI.**	**N/A**
**Section 7: Study Results**		
**7a. Outcomes of PPI**	**Report the results of PPI in the study, including both positive and negative outcomes.**	**Pages 18-26**
**7b. Impacts of PPI**	**Report the positive and negative impacts that PPI has had on the research, the individuals involved (including patients and researchers), and wider impacts.**	**Pages 18-26**
**7c. Context of PPI**	**Report the influence of any contextual factors (e.g., availability of funding, policy) that enabled or hindered the impact of PPI.**	**Page 31 (in discussion)**
**7d. Process of PPI**	**Report the influence of any process factors (e.g., how users were involved), that enabled or hindered the impact of PPI.**	**Pages 31 (in discussion)**
**7e. Theory development**	**i) Report any conceptual or theoretical development in PPI that have emerged from the study.**	**N/A**
	**ii) If applicable, report evaluation of theoretical models.**	**N/A**

**7f. Measurement**	**If applicable, report all aspects of instrument development and testing (e.g., validity, reliability, feasibility, acceptability, responsiveness, interpretability, appropriateness, precision).**	**N/A**
**7g. Economic assessment**	**Report any information on the economic cost or benefit of PPI.**	**N/A**
**Section 8: Discussion and Conclusions**		
**8a. Outcomes**	**Comment on how PPI influenced the study overall. Describe positive and negative effects.**	**Page 31**
**8b. Impacts**	**Comment on the different impacts of PPI identified in this study and how they contribute to new knowledge.**	**Pages 26-31**
**8c. Definition**	**Comment on the definition of PPI used in the study (reported in the Background section) and whether or not you would suggest any changes.**	**N/A**
**8d. Theoretical underpinnings**	**Comment on any way your study adds to the theoretical development of PPI.**	**N/A**
**8e. Context**	**Comment on how context factors influenced PPI in the study.**	**Page 31**
**8f. Process**	**Comment on how process factors influenced PPI in the study.**	**Pages 31**
**8g. Measurement and capture of PPI impact**	**If applicable, comment on how well PPI impact was evaluated or measured in the study.**	**Page 33**
**8h. Economic information**	**If applicable, discuss any aspects of economic cost or benefit of PPI, particularly any suggestions for future economic modelling.**	**N/A**
**8i. Reflections/ critical perspective**	**Comment critically on the study, reflecting on the things that went well and those that did not, so others can learn from this study.**	**Pages 31-34**
